# Iron-deficiency Anemia Causes an Ischemic Stroke in a Young Man

**DOI:** 10.7759/cureus.4218

**Published:** 2019-03-11

**Authors:** Rose Fluss, Liridon Zguri, Ralph Rahme, Ilmana Fulger

**Affiliations:** 1 Neurosurgery, St. Barnabas Hospital Health System, Bronx, USA; 2 Internal Medicine, St. Barnabas Hospital Health System, Bronx, USA; 3 Hematology-Oncology, St. Barnabas Hospital Health System, Bronx, USA

**Keywords:** ischemic stroke, iron-deficiency anemia, periaqueductal gray

## Abstract

Iron deficiency anemia is an unusual etiology of ischemic stroke in the adult population. Only a few case reports exist describing this rare cause of cerebrovascular accident due to low circulating levels of oxygen. To the best of our knowledge, we believe we have described here the first patient presenting with an ischemic stroke secondary to severe anemia without an obvious source of blood loss, evidence of thrombus formation, or associated thrombocytosis.

A 28-year-old man presented to our institution, complaining of severe fatigue and was admitted under the pretext of dizziness, blurred vision, esotropia, diffuse retinal hemorrhage, and Roth spots found in the left eye. Laboratory investigations reported a hemoglobin level of 1.12 g/dL (13.5-17.5 g/dl) and thrombocytopenia with a platelet count of 16,000/ µL (150,000-450,000). Concomitant magnetic resonance imaging (MRI) of the brain revealed a right 4 mm periaqueductal lesion. After several red blood cell transfusions, the patient hereafter underwent an improvement in his clinical symptoms. Given no past medical history of anemia or thrombocytopenia, an extensive workup was prompted, but only microcytic anemia likely due to iron deficiency was found.

Regarding a stroke in the young adult population, a differential beyond atherosclerosis and hypertension must be considered. Coagulopathies, vasculitis, sickle cell anemia, cerebral venous thrombosis, cocaine abuse, and systemic hypoperfusion are less common, but well-documented, causes of ischemia. Although infrequently mentioned, we demonstrate here a real possibility of severe iron deficiency anemia causing ischemic stroke in the brainstem of a young man. While a thorough neurologic evaluation is not often considered in patients presenting with signs and symptoms of severe anemia, vigilance regarding focal neurologic deficits should prompt suspicion for ischemic stroke in patients with significantly low hemoglobin levels.

## Introduction

Severe anemia is an extremely rare cause of ischemic stroke. Previous investigations determined profound iron deficiency anemia to be the principal source of anoxic injury to the brain. However, these former reports have always been associated with a large known source of bleeding, causing systemic hypoperfusion or a coexisting thrombus or thrombocytosis with evidence of resultant emboli [1-5]. Presented here is a case of a 28-year-old male with evidence of midbrain ischemia found on magnetic resonance imaging (MRI) and with a workup that displayed no evidence for a source of emboli, large loss of blood volume, or underlying genetic predisposition for blood dyscrasia. Additionally, the details of this patient are discussed, as well as the diagnostic tests and the clinical reasoning behind making the diagnosis of iron deficiency anemia as the cause of ischemic stroke in this young man.

## Case presentation

A 28-year-old male came to the emergency department, complaining of profound fatigue, chest pain on exertion, dizziness, diplopia, and headaches. He endorsed heavy marijuana and alcohol use. Physical exam elicited a thin man, poor dental hygiene, tachycardia, and a soft apical murmur.

**Figure 1:**
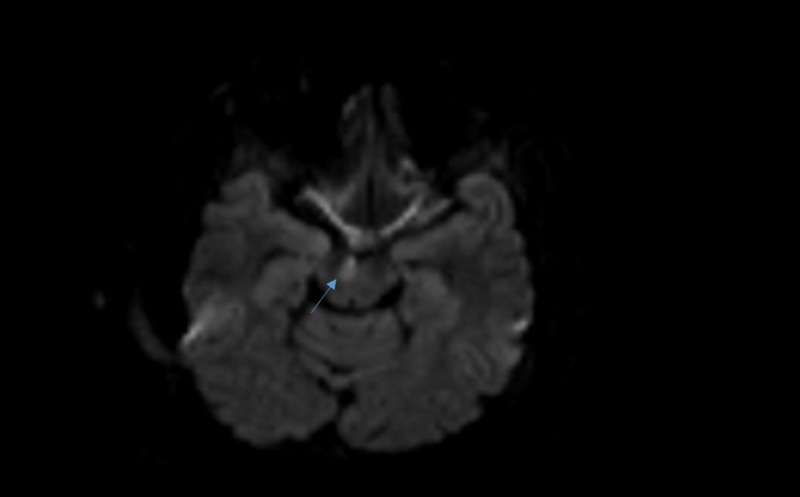
Magnetic resonance imaging showing evidence of a stroke in the right periaqueductal gray

Ophthalmic examination revealed esotropia of the left eye, diffuse retinal hemorrhages, and Roth spots. An electrocardiogram was read as normal. MRI revealed evidence of a stroke with a 4 mm lesion of the right periaqueductal gray (PAG) (Figure 1). A complete blood count (CBC) showed a hemoglobin level of 1.12 g/dl (normal: 13.5-17.5 g/dl) and a platelet count of 16,000/µL (normal: 150,000-450,000). Repeat CBCs two and six hours later were 1.28 g/dl and 1.73 g/dl, respectively, while his repeat platelet count was 7,000/µL. The patient was emergently transfused, totaling six units of packed red blood cells (pRBC) and one unit of platelets over the next three days. He was also started on judicious iron supplementation due to a peripheral blood smear displaying severely microcytic and hypochromic red blood cells. After three units of pRBC, his presenting clinical symptoms, including his focal neurologic deficit of esotropia, began resolving.

## Discussion

Reported here is the case of a 28-year-old man who suffered from an ischemic stroke due to severe anemia. Based upon his staggeringly low red blood cell counts (hemoglobin level of 1.12 g/dl), and otherwise normal workup for stroke in a man this age, we presume severe anemia was the cause of his stroke. Since his mean corpuscular volume was low (58.6 fL) and his red blood cells were hypochromic, we believe iron deficiency was the source for his anemia.

Similarly to the elderly population, atherosclerosis is often cited as the predominant cause of ischemic stroke in young adults [6]. However, since our patient had a healthy body mass index (BMI) and normal lipid levels, and was absent of other vascular risk factors, including diabetes and hypertension, we chose to explore less-common potential causes of his stroke. Given his physical exam findings of poor dental hygiene and Roth spots, septic emboli secondary to infective endocarditis was our initial leading diagnosis. However, blood cultures were negative and the echocardiogram did not show vegetations (with only a possible mitral valve prolapse). His urine toxicology screened positive for only tetrahydrocannabinol (THC), effectively ruling out cocaine abuse as the cause of his stroke. A colonoscopy revealed no source of bleeding, excluding a gastrointestinal bleed as a cause of systemic hypoperfusion and origin of his iron deficiency anemia. An abdominal ultrasound exhibited no evidence hepatic venous thrombosis. This finding, along with a normal haptoglobin and normal levels of CD 55 and 59 helped us exclude thrombosis due to paroxysmal nocturnal hemoglobinuria. Additionally, he was negative for Factor V Leiden mutation and lupus anticoagulant for excluding an inherited hypercoagulable condition and antiphospholipid syndrome. His hemoglobin electrophoresis eliminated the possibility of sickle cell anemia. An inflammatory vasculitis would likely present with more diffuse lesions across the vasculature and not a single lesion like in our patient. Bone marrow aspirate and biopsy failed to reveal any myeloproliferative disorder such as leukemia. Additional tests were performed in light of his thrombocytopenia, but both human immunodeficiency virus (HIV) and the hepatitis B and C panel was negative. This led us to believe that his extremely low hemoglobin was the source of ischemia and that perhaps his heavy alcohol consumption was the culprit of his iron deficiency.

The literature describes very few case reports of patients who have experienced ischemic infarctions solely as a result of anemia. Of those, Gopalratnam et al. detail a 20-year-old female with a history of menorrhagia and syncope showing ischemic infarctions on imaging and an acute presentation of left-sided weakness [1]. Unlike the patient we present here, laboratory tests in that case revealed an elevated platelet count of 564,000/ µL. Similarly, Naito et al. provide two accounts of 42-year-old females with a history of menorrhagia presenting with hemiparesis as a result of bleeding uterine fibroids [2]. Both cases had normal platelet counts of 260,000/μL and 429,000/μL. The last case detailed in the literature describes a 47-year-old woman, also with a history of menorrhagia, who exhibited mild dysarthria, upper extremity weakness, and thrombocytosis with a platelet count of 512,000/μL [1]. There also exists in the literature five reported cases of severe anemia causing a thrombus formation [2,5]. All cases revealed a carotid artery thrombus found on MRI. Only in one case did a patient present with normal platelet counts and without a reactive thrombocytosis [5]. These previous studies propose one of the following two hypotheses to explain cerebrovascular accidents associated with anemia:

1. Low circulating oxygen levels motivate an increase in cerebral blood flow, which may provoke endothelial damage and thus cause thrombus formation and subsequent reactive thrombocytosis [2,5].

2. Anemic hypoxia acts much like systemic hypoperfusion in that it leaves major watershed areas under-perfused and susceptible to ischemic injury [1,3-4].

Whereas the latter mechanism entails low oxygen transport and is reinforced by their patients suffering from infarctions in major watershed areas [1,3-4], our patient suffered from a stroke in the periaqueductal gray matter (PAG). Although not a watershed area, the PAG is a highly active metabolic brain region that may have suffered from the insult of low circulating oxygen levels in our patient. While best known for the inhibition of pain, the PAG has been implicated in micturition control, migraines, vertical ophthalmoplegia, and, in this case, our patient presenting with esotropia [7].

## Conclusions

To the best of our knowledge, this is the first case describing a young man presenting with a dangerously low hemoglobin level of 1.12 g/dl and showing evidence of a brainstem stroke in the periaqueductal gray. Upon numerous red blood cell transfusions, the patient clinically improved and underwent a reversal of his neurologic deficit of esotropia. The results of several diagnostic tests led us to believe the etiology of his stroke was caused by his iron deficiency anemia. Previously documented cases of stroke caused by severely low hemoglobin similarly saw the resolution of neurologic deficits upon treatment of the anemia. We, therefore, advise that a thorough neurologic evaluation should be considered in all patients presenting with signs and symptoms of severe anemia. Vigilance regarding hemoglobin levels and prompt treatment of anemia may potentially prevent a cerebrovascular accident and the adverse sequela associated with these events.
